# Impact of Consumer Wearables Data on Pediatric Surgery Clinicians’ Management: Multi-Institutional Scenario-Based Usability Study

**DOI:** 10.2196/58663

**Published:** 2024-11-12

**Authors:** Michela Carter, Samuel C Linton, Suhail Zeineddin, J Benjamin Pitt, Christopher De Boer, Angie Figueroa, Ankush Gosain, David Lanning, Aaron Lesher, Saleem Islam, Chethan Sathya, Jane L Holl, Hassan MK Ghomrawi, Fizan Abdullah

**Affiliations:** 1 Division of Pediatric Surgery, Department of Surgery Ann and Robert H Lurie Children's Hospital of Chicago Northwestern University Feinberg School of Medicine Chicago, IL United States; 2 Division of Pediatric Surgery, Department of Surgery Children’s Hospital Colorado University of Colorado School of Medicine Aurora, CO United States; 3 Division of Pediatric Surgery, Department of Surgery Children’s Hospital of Richmond at Virginia Commonwealth University Richmond, VA United States; 4 Division of Pediatric Surgery, Department of Surgery Medical University of South Carolina Charleston, SC United States; 5 Division of Pediatric Surgery, Department of Surgery University of Florida College of Medicine Gainesville, FL United States; 6 Division of Pediatric Surgery, Department of Surgery Cohen Children’s Medical Center Donald and Barbara Zucker School of Medicine at Hofstra/Northwell New Hyde Park, NY United States; 7 Department of Neurology and Center for Healthcare Delivery Science and Innovation University of Chicago Chicago, IL United States; 8 Department of Orthopaedic Surgery Heersink School of Medicine University of Alabama at Birmingham Birmingham, AL United States

**Keywords:** postoperative care, telehealth, consultation, remote, appendectomy, pediatric hospital, children, wearable device, minimally invasive surgery, pediatric surgery, remote simulation study

## Abstract

**Background:**

At present, parents lack objective methods to evaluate their child’s postoperative recovery following discharge from the hospital. As a result, clinicians are dependent upon a parent’s subjective assessment of the child’s health status and the child’s ability to communicate their symptoms. This subjective nature of home monitoring contributes to unnecessary emergency department (ED) use as well as delays in treatment. However, the integration of data remotely collected using a consumer wearable device has the potential to provide clinicians with objective metrics for postoperative patients to facilitate informed longitudinal, remote assessment.

**Objective:**

This multi-institutional study aimed to evaluate the impact of adding actual and simulated objective recovery data that were collected remotely using a consumer wearable device to simulated postoperative telephone encounters on clinicians’ management.

**Methods:**

In total, 3 simulated telephone scenarios of patients after an appendectomy were presented to clinicians at 5 children’s hospitals. Each scenario was then supplemented with wearable data concerning or reassuring against a postoperative complication. Clinicians rated their likelihood of ED referral before and after the addition of wearable data to evaluate if it changed their recommendation. Clinicians reported confidence in their decision-making.

**Results:**

In total, 34 clinicians participated. Compared with the scenario alone, the addition of reassuring wearable data resulted in a decreased likelihood of ED referral for all 3 scenarios (*P*<.01). When presented with concerning wearable data, there was an increased likelihood of ED referral for 1 of 3 scenarios (*P*=.72, *P*=.17, and *P*<.001). At the institutional level, there was no difference between the 5 institutions in how the wearable data changed the likelihood of ED referral for all 3 scenarios. With the addition of wearable data, 76% (19/25) to 88% (21/24 and 22/25) of clinicians reported increased confidence in their recommendations.

**Conclusions:**

The addition of wearable data to simulated telephone scenarios for postdischarge patients who underwent pediatric surgery impacted clinicians’ remote patient management at 5 pediatric institutions and increased clinician confidence. Wearable devices are capable of providing real-time measures of recovery, which can be used as a postoperative monitoring tool to reduce delays in care and avoidable health care use.

## Introduction

When children are discharged from the hospital after surgery, clinicians depend on caregivers’ surveillance of the patient and analysis of their recovery to initiate communication with the health care team. When a caregiver contacts the surgical team with concerns, clinicians rely on the caregiver’s narrative of the patient’s experience after discharge in order to triage the patient. Currently, caregivers lack objective methods to evaluate recovery after discharge. As a result, they are dependent upon their subjective assessment of the child’s well-being and the child’s ability to communicate their symptoms. It has been shown that the subjective nature of home monitoring contributes to both avoidable health care use and delays in treatment [1-4].

In the United States, laparoscopic appendectomy is the most common inpatient procedure in children, with approximately 80,000 to 100,000 performed annually [5]. Nearly 20% of appendectomies result in emergency department (ED) visits or readmissions within 90 days postoperatively, and greater than 40% of these ED presentations are potentially avoidable [6]. Clinician access to objective recovery data offers the potential for improved patient triage in the postoperative setting and would serve to reduce delays in care and unnecessary health care use. Consumer wearable devices, for example, Fitbit (Google), have the ability to provide continuous objective measurements of recovery, which include heart rate, step count, and sleep assessment (ie, “wearable data”). Furthermore, these data can be made available to clinicians in near real time. With such features, wearable devices have the potential to assist clinicians in the remote evaluation and triage of postoperative patients after discharge [7-10].

Within our institution, we previously demonstrated that the addition of wearable data to simulated scenarios of unplanned postoperative episodes of health care use impacted pediatric surgery clinicians’ decision-making, including a significant difference in the likelihood of recommending immediate presentation to the ED and increased confidence in clinicians’ decision-making [10]. However, the results may not be generalizable to other institutions that are not as familiar with the use of wearable devices in the postoperative setting. Therefore, the objective of this multi-institutional study was to evaluate whether the addition of actual and simulated objective data derived from a consumer-grade wearable device to telephone encounters derived from actual postoperative patient encounters impacted the decision-making of a diverse cohort of pediatric surgery clinicians when presented in a simulation environment.

## Methods

### Study Design

To evaluate the clinical use of wearable data, we presented 3 simulated, postdischarge telephone scenarios to pediatric surgery clinicians. The 3 scenarios were based on actual patients who underwent laparoscopic appendectomy for acute appendicitis at an urban, tertiary children’s hospital. All 3 patients had worn the Fitbit Inspire, a consumer-grade wearable device, for 21 days after surgery as part of a previous study [8]. Surgeon authors (SL, CDB, and FA) selected these 3 patients to feature the most common postoperative complications following laparoscopic appendectomy, surgical site infections, and clinical scenarios, which could have been clarified with the addition of wearable data [11]. The three scenarios presented were as follows: (1) a 13-year-old female patient who underwent laparoscopic appendectomy for complicated appendicitis, and on a postoperative day 7, her caregiver called reporting 2 days of abdominal pain, loose stools, and incisional drainage; (2) a 10-year-old female patient who underwent laparoscopic appendectomy for simple appendicitis, and on postoperative day 3, her caregiver called with report of 2 days of fevers, abdominal pain, and periumbilical erythema; and (3) a 9-year-old male patient who underwent laparoscopic appendectomy for complicated appendicitis, and on postoperative day 10, his caregiver called with report of 2 days of purulent drainage from one of his surgical incisions.

Daily step counts and heart rate data were measured by Fitbit and recorded in Fitabase, a third-party, Health Insurance Portability and Accountability Act (HIPAA)–compliant database, designed to track data provided by an enrolled Fitbit device. The Fitbit data, in addition to information from the patient’s electronic medical record, including actual documented encounters between the caregiver and pediatric surgery clinicians and the documented descriptions of the patient’s symptoms as reported by the caregiver, were used to generate the simulated telephone scenarios. For each scenario, the patient’s wearable data were used to create a daily heart rate graph and a daily step count graph, both of which included data from postoperative day 1 through the date of the telephone encounter. In addition, the patient’s average, minimum, and maximum heart rate in the 5 minutes, 1 hour, 4 hours, and 24 hours leading up to the encounter were displayed in a table. Using Fitbit data collected during our previously published study, the age- and sex-adjusted step counts collected from patients with an uncomplicated postoperative course after the same surgery were included as a normative reference for the clinician evaluating the patient’s scenario [8].

The study team evaluated the patient’s actual data at the time of the telephone encounter and classified it as concerning if the patient’s heart rate was elevated and physical activity reduced relative to the normative reference data. Contrarily, wearable data were classified as reassuring if the heart rate and physical activity were approximate to the normative reference data. The study team then created simulated wearable data for each scenario that were opposite to the actual data, that is, simulated wearable data were concerning (elevated heart rate and low step count) when the patient’s actual wearable data were reassuring (heart rate and step count within normal range for age). The source of the wearable data and the classification as concerning or reassuring were not shared with the clinicians who participated in the study. Representative concerning and reassuring wearable data for the 24 hours preceding the time of encounter for the 3 scenarios are demonstrated in Table 1.

**Table 1:** Concerning and reassuring wearable data for the 24 hours preceding the simulated telephone encounter for scenarios 1-3.

Patient and wearable data			Concerning		Reassuring
**Scenario 1: 13-year-old female patient, POD^a^ 7**					
	Average heart rate (bpm)	86		86	
	Minimum heart rate (bpm)	63		63	
	Maximum heart rate (bpm)	121		103	
	Step count	1100		6100	
**Scenario 2: 10-year-old female patient, POD 3**					
	Average heart rate (bpm)	80		80	
	Minimum heart rate (bpm)	60		60	
	Maximum heart rate (bpm)	142		105	
	Step count	650		5100	
**Scenario 3: 9-year-old male patient, POD 10**					
	Average heart rate (bpm)	109		92	
	Minimum heart rate (bpm)	93		78	
	Maximum heart rate (bpm)	144		113	
	Step count	2100		5050	

^a^POD: postoperative day.

A total of 5 pediatric institutions, located throughout the United States, elected to participate in this study. The institutions that participated were diverse in practice settings; however, all were associated with an academic institution. Pediatric surgery clinicians, including attending surgeons, resident surgeons, and advanced practice providers, were recruited from the 5 participating institutions. Poll Everywhere audience response software was used for survey participation. At the start of the survey, the participants were oriented to wearable data from a patient with an uncomplicated postoperative course following laparoscopic appendectomy. The 3 telephone scenarios were then presented to the clinician participants in 3 formats. First, the scenario was presented without wearable data and participants were asked to triage the patient and determine the urgency for follow-up care, including seeking care immediately, prescribing a medication with outpatient follow-up, outpatient follow-up alone, and providing reassurance without the need for follow-up. Clinicians were then asked to rate their “likelihood to recommend the patient present to the ED immediately” using a 10-point Likert scale, with 1 representing “not at all likely to recommend ED presentation” and 10 representing “definitely would recommend ED presentation.”

The participants were then shown the telephone scenario with concerning and reassuring wearable data in random sequence and without revealing the classification to the respondents. Participants were asked about their likelihood of recommending ED presentations using the same 10-point Likert scale for both sets of wearable data. They were then asked to report if the wearable data increased their confidence in their recommendation and, if provided the wearable data alone, they would initiate contact with the patient and caregiver to assess their recovery. Participants were only offered the opportunity to respond to each multiple-choice question once. In total, the participants were asked to score their likelihood of recommending ED presentation for all 3 scenarios without wearable data, with concerning wearable data, and with reassuring wearable data for a total of 9 recommendations for ED presentation.

### Statistical Analysis

Survey responses were determined to be nonparametric by Shapiro-Wilk testing. Descriptive analyses were performed and included frequencies of response and median (IQR). Wilcoxon rank sum test was performed comparing the clinician’s recommendation for ED presentation without wearable data to their recommendation with concerning wearable data and reassuring wearable data. The likelihood of ED referral without wearable data was then subtracted from the likelihood of ED referral with wearable data to evaluate how a clinicians’ management recommendation changed. A positive change was consistent with an increased likelihood of ED referral while a negative change was consistent with a decreased likelihood of ED referral. No difference indicated no change in the likelihood of ED referral. To evaluate for institutional variation, the proportion of survey respondents at each institution who were more likely to refer, were less likely to refer, and did not change their likelihood of ED referral with the addition of wearable data was determined for each scenario and compared by Fisher exact test. Statistical significance was defined as *P*<.05.

### Ethical Considerations

This study received ethics exemption from Ann and Robert H Lurie Children's Hospital (IRB #2022-5553). The relevant guideline which supports exemption status is based on Lurie guidelines: the disclosure of the participants’ responses outside the research would not reasonably place the participants at risk of criminal or civil liability or be damaging to the participants’ financial standing, employability, educational advancement, or reputation.

## Results

### Study Participants

In total, 34 clinicians voluntarily participated in the study (Table 2). Site 3 contributed the greatest complement with 12 participants, accounting for 35% (12/34) of the study cohort. The smallest contributing site was site 1 with 4 participants, accounting for 12% (4/34) of the study cohort. In total, 65% (22/34) of the participants were attending surgeons, 15% (5/34) were advanced practice providers, 15% (5/34) were surgery residents, and 6% (2/34) did not report clinician type. Response rates ranged from 68% (23/34) to 85% (29/34) responses per survey question.

**Table 2:** Survey participants by institution and clinician type.

Institution	Attending, n (%)^a^	Advanced practice providers, n (%)^a^	Resident, n (%)^a^	Not reported, n (%)^a^	Total (N=34), n (%)
Site 1	4 (100)	0 (0)	0 (0)	0 (0)	4 (12)
Site 2	4 (67)	2 (33)	0 (0)	0 (0)	6 (18)
Site 3	4 (33)	1 (8)	5 (42)	2 (17)	12 (35)
Site 4	5 (100)	0 (0)	0 (0)	0 (0)	5 (15)
Site 5	5 (71)	2 (29)	0 (0)	0 (0)	7 (21)
All sites	22 (65)	5 (15)	5 (15)	2 (6)	34 (100)

^a^Percentage values are calculated using the values in the “Total” column as the denominators.

### Scenario 1

When scenario 1 was presented without wearable data, 17 (61%) of 28 respondents recommended outpatient follow-up, while 10 (36%) recommended they seek care immediately and 1 (4%) recommended reassurance without follow-up. When asked to rank the likelihood of recommending ED presentation, the median recommendation was 5 (IQR 3-7). When presented with reassuring wearable data, the median recommendation for ED presentation was 2 (IQR 1-3) with a median change from when no wearable data were available of –2 (IQR –4 to –1; *P*<.001). ED referral was less likely for 24 (86%) of 28 respondents in response to the reassuring wearable data while 2 (7%) did not change their recommendation and 2 (7%) were more likely to recommend ED presentation. In total, 22 (85%) of 26 respondents reported increased confidence in their recommendation with the addition of the reassuring wearable data, while 6 (24%) of 25 reported that if they had been presented with the reassuring wearable data alone, they would have initiated contact with the patient or caregiver in order to evaluate for a postoperative complication.

**Figure 1:**
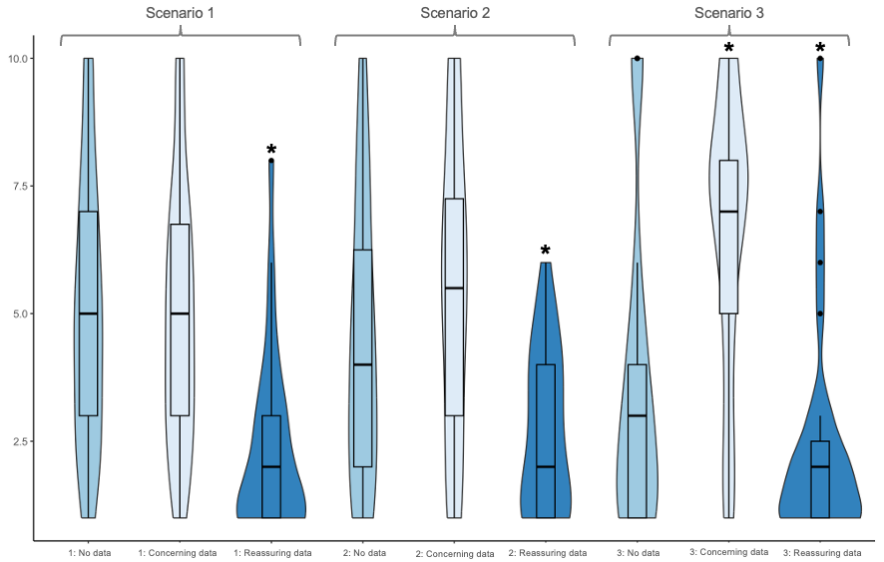
Recommendation for emergency department presentation provided by pediatric surgery clinicians at 5 institutions when presented with 3 simulated telephone scenarios: (1) without wearable data, (2) with concerning wearable data, and (3) with reassuring wearable data. Likelihood of emergency department referral reported on a 10-point Likert Scale with 1 representing “Not at all likely” and 10 representing “Definitely”. *Significant change by Wilcoxon rank sum test.

**Figure 2:**
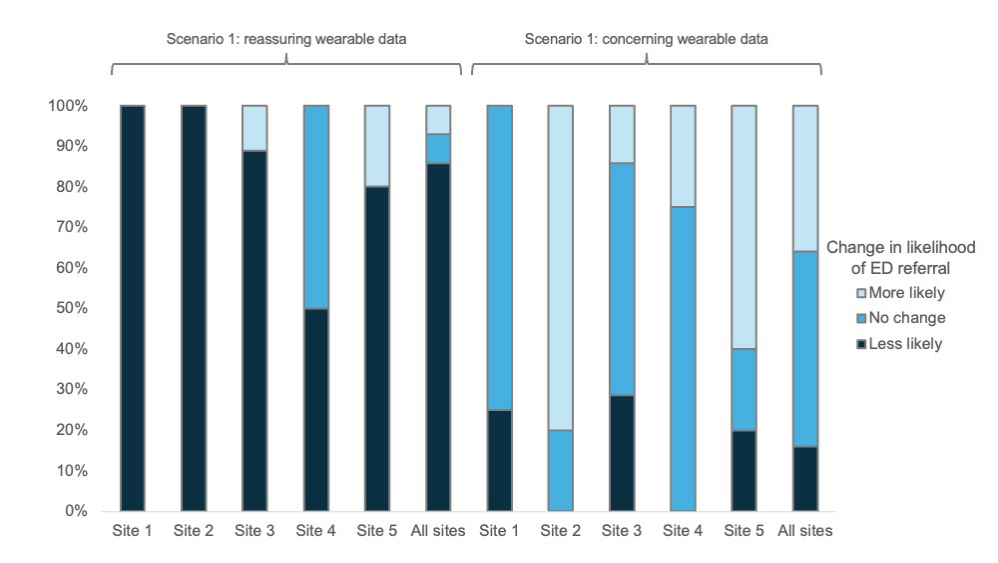
How the likelihood of emergency department referral changed at each institution with the addition of reassuring and concerning wearable data to scenario 1. ED: emergency department.

When the scenario was presented with concerning wearable data, the median recommendation for ED presentation was 5 (IQR 3-7) with a median change of 0 (IQR 0-2; *P*=.72). A total of 9 (36%) of 25 respondents were more likely to recommend ED referral in response to the concerning wearable data, while 12 (48%) had no change in their recommendation and 4 (16%) were less likely to recommend ED referral. In total, 21 (88%) of 24 participants reported increased confidence in their recommendation, and 22 (85%) of 26 reported they would reach out to the patient or caregiver if presented the wearable data alone. Survey responses for scenario 1 are summarized in Table 3 and Figure 1. Response to the addition of reassuring and concerning wearable data by institutions is demonstrated in Figure 2. There was no difference between institutions in how they responded to the addition of reassuring (*P*=.10) or concerning wearable data (*P*=.18).

**Table 3:** Simulated remote management recommendations from pediatric surgery clinicians at 5 institutions in response to telephone scenario 1 presented without wearable data, then with reassuring and concerning wearable data. Scenario 1: 13-year-old female patient on postoperative day 7 following laparoscopic appendectomy for complicated appendicitis, now with 2 days of abdominal pain, loose stools, and incisional drainage.

		No wearable data	Reassuring wearable data	Concerning wearable data
**Initial recommendation (n=28), n (%)**				
	Seek care immediately	10 (36)	—^a^	—
	Prescription and outpatient follow-up	0 (0)	—	—
	Outpatient follow-up	17 (61)	—	—
	Reassurance and no follow-up	1 (4)	—	—
**Likelihood of ED^b^ referral, median (IQR)**		5 (3 to 7)	2 (1 to 3)	5 (3 to 7)
	Change in the likelihood of ED referral, median (IQR)	—	–2 (–4 to –1)	0 (0 to 2)
	*P* value	—	<.001	.72
**Increased confidence, n/N (%)**		—	22/26 (85)	21/24 (88)
**Would reach out to patient or caregiver, n/N (%)**		—	6/25 (24)	22/26 (85)

^a^Not applicable.

^b^ED: emergency department.

### Scenario 2

When scenario 2 was presented without wearable data, 14 (50%) of 28 respondents recommended outpatient follow-up, while 7 (25%) recommended a prescription and outpatient follow-up and 7 (25%) recommended the patient should seek care immediately. The median likelihood of recommending ED presentation was 4 (IQR 2-6.75). When reassuring wearable data was presented with the patient scenario, the median likelihood of recommendation for ED presentation decreased to 2 (IQR 1-4). This represented a median change in score of –1 (IQR –2.5 to 0; *P*<.001). ED referral was less likely for 16 (62%) of 26 respondents in response to the reassuring wearable data, while 7 (27%) did not change and 3 (12%) were more likely to recommend ED presentation. In total, 23 (85%) of 27 respondents reported increased confidence in their recommendation when the reassuring wearable data were added. Only 7 (30%) of 23 reported they would initiate contact with the patient or caregiver in response to the reassuring wearable data alone.

**Figure 3:**
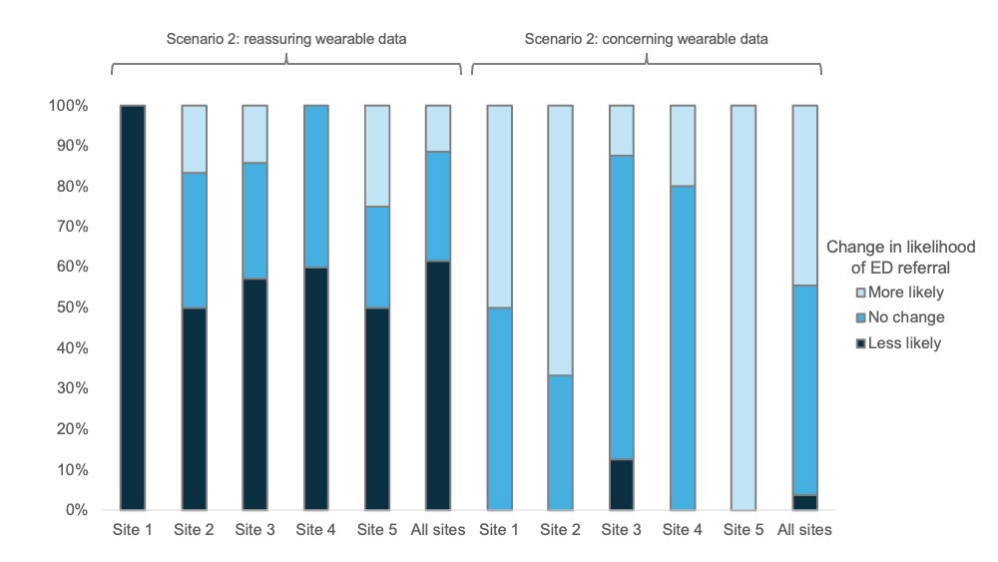
How the likelihood of emergency department referral changed at each institution with the addition of reassuring and concerning wearable data to scenario 2. ED: emergency department.

When concerning wearable data were presented with the scenario, the median recommendation for ED presentation was 5.5 (IQR 3-7.75) representing a median change of 0 (IQR 0-2; *P*=.17). With the addition of concerning wearable data, 12 (44%) of 27 respondents were more likely to recommend ED referral, while 14 (52%) had no change in their recommendation and 1 (4%) was less likely to recommend ED referral. In addition, 19 (76%) of 25 reported increased confidence with this recommendation, and 18 (80%) of 25 reported they would reach out to the patient or caregiver if presented the concerning wearable data alone. Survey responses for scenario 2 are summarized in Table 4 and Figure 1. Response to the addition of reassuring and concerning wearable data for scenario 2 by institution is demonstrated in Figure 3. There was no significant difference between institutions in their response to the addition of reassuring (*P*=.90) or concerning wearable data (*P*=.05).

**Table 4:** Simulated remote management recommendations from pediatric surgery clinicians at 5 institutions in response to telephone scenario 2 presented without wearable data, then with reassuring and concerning wearable data. Scenario 2: 10-year-old female patient on postoperative day 3 following laparoscopic appendectomy for simple appendicitis, now with 2 days of fevers, abdominal pain, and periumbilical erythema.

		No wearable data	Reassuring wearable data	Concerning wearable data
**Initial recommendation (n=28), n (%)**				
	Seek care immediately	7 (25)	—^a^	—
	Prescription and outpatient follow-up	7 (25)	—	—
	Outpatient follow-up	14 (50)	—	—
	Reassurance and no follow-up	0 (0)	—	—
**Likelihood of ED^b^ referral, median (IQR)**		4 (2 to 6.75)	2 (1 to 4)	5.5 (3 to 7.75)
	Change in likelihood of ED referral, median (IQR)	—	–1 (–2.5 to 0)	0 (0 to 2)
	*P* value	—	<.001	.17
**Increased confidence, n/N (%)**		—	23/27 (85)	19/25 (76)
**Would reach out to patient or caregiver, n/N (%)**		—	7/23 (30)	20/25 (80)

^a^Not applicable.

^b^ED: emergency department.

### Scenario 3

When scenario 3 was presented without wearable data, 18 (64%) of 28 recommended outpatient follow-up, while 6 (21%) recommended the patient seek care immediately and 4 (14%) recommended a prescription with outpatient follow-up. When asked about the likelihood of recommending ED presentation, the median score was 3 (IQR 1-4.5). When reassuring wearable data were added, the median recommendation dropped to 2 (IQR 1-3) representing a median decrease in recommendation of 0 (IQR –2 to 0; *P*=.002). ED referral was less likely for 13 (48%) of 27 in response to the reassuring wearable data, while 13 (48%) did not change and 1 (4%) was more likely to recommend ED presentation. In total, 23 (85%) of 27 clinicians reported increased confidence in their recommendation when the reassuring wearable data were added, while 6 (24%) of 25 reported they would reach out to the patient or caregiver if presented the reassuring wearable data alone.

**Figure 4:**
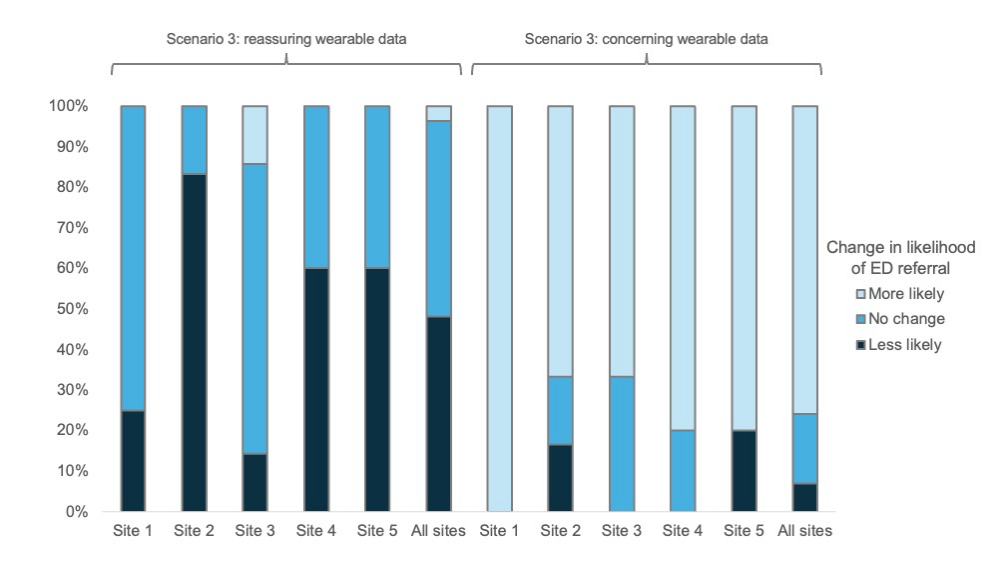
How the likelihood of emergency department referral changed at each institution with the addition of reassuring and concerning wearable data to scenario 3. ED: emergency department.

When presented concerning wearable data, the median recommendation for presentation to the ED increased to 7 (IQR 5-8), a median increase of 3 (IQR 0.5-5; *P*<.001). With the addition of concerning wearable data, 22 (76%) of 29 respondents were more likely to recommend ED referral, while 5 (17%) had no change in their recommendation and 2 (7%) were less likely to recommend ED referral. In total, 22 (88%) of 25 reported increased confidence in their recommendation when concerning wearable data were present. In addition, 23 (96%) of 24 reported they would initiate contact with the patient or caregiver if presented the concerning wearable data alone. Survey responses for scenario 3 are summarized in Table 5 and Figure 1. Institutional response to the addition of reassuring and concerning wearable data for scenario 3 is demonstrated in Figure 4. There was no significant difference between institutions in their response to the addition of reassuring (*P*=.20) or concerning wearable data (*P*=.57).

**Table 5:** Simulated remote management recommendations from pediatric surgery clinicians at 5 institutions in response to telephone scenario 3 presented without wearable data, then with reassuring and concerning wearable data. Scenario 3: a 9-year-old male patient on postoperative day 10 following laparoscopic appendectomy for complicated appendicitis, now with 2 days of purulent drainage from the surgical port site.

			No wearable data		Reassuring wearable data		Concerning wearable data
**Initial recommendation (n=28), n (%)**							
	Seek care immediately	6 (21)		—^a^		—	
	Prescription and outpatient follow-up	4 (14)		—		—	
	Outpatient follow-up	18 (64)		—		—	
	Reassurance and no follow-up	0 (0)		—		—	
**Likelihood of ED^b^ referral, median (IQR)**			3 (1 to 4.5)		2 (1 to 3)		7 (5 to 8)
	Change in likelihood of ED referral, median (IQR)	—		0 (–2 to 0)		3 (0.5 to 5)	
	*P* value	—		.002		<.001	
**Increased confidence, n/N (%)**			—		23/27 (85)		22/25 (88)
**Would reach out to patient or caregiver, n/N (%)**			—		6/25 (24)		23/24 (96)

^a^Not applicable.

^b^ED: emergency department.

## Discussion

### Principal Findings

This study investigated the potential impact that postoperative objective measures of recovery collected by a consumer-grade wearable device, the Fitbit, may have on the decision-making of pediatric surgery clinicians from 5 children’s hospitals in the United States. We found significant changes in recommendation for ED presentation when simulated telephone scenarios were supplemented with heart rate and step count data derived from Fitbit. Clinicians reported increased confidence with their decision-making when supplemented with wearable data. In addition, the majority of clinicians reported they would initiate contact with the patient and caregiver if they were presented with concerning wearable data in isolation. How wearable data impacted clinicians’ likelihood of ED referral did not differ between institutions. These findings support consumer wearables as a generalizable clinical tool and provide further impetus for their adoption as a low-cost and efficient postoperative postdischarge remote monitoring technology with the potential to decrease the burden of unnecessary health care use and delays in seeking care.

Our study demonstrates that when clinicians are supplied with objective data from a wearable device, they are able to interpret these data and incorporate them into their decision-making with significant changes in their recommendations for ED presentation compared with when no wearable data were provided. In the current practice model, a “worst-case” mindset is assumed. The clinician is blinded to any objective measure of recovery and is solely dependent on the subjective narrative provided to them by the caregiver and patient. Patient safety and the medicolegal system necessitate this practice; however, it perpetuates health care saturation and associated costs as it often results in referral for an in-person evaluation. The addition of objective data has the potential to reassure the clinician or reinforce, and even augment, clinical concern. For example, in scenarios 1 and 2, there was no change in recommendation for ED presentation when concerning wearable data were added; therefore, the subjective information alone was concerning and the addition of objective data only strengthened confidence in this recommendation. However, when reassuring wearable data were supplied, the clinicians were significantly less likely to recommend ED presentation. As the subjective information for these scenarios did not change, this highlights the use of objective measures of recovery and their value in clinical decision-making. Alternatively, when scenario 3 was presented with concerning wearable data, the clinicians’ recommendation for ED presentation significantly increased; therefore, augmenting clinical concern for a postoperative complication. This demonstrates how delays in care may be avoided with the addition of wearable data.

Not only did the wearable data change the clinicians’ assessment of postoperative, postdischarge patients, but the data also gave the clinicians more confidence in their decisions. Greater than three-fourths of clinicians reported increased confidence in their recommendations when wearable data were added for all scenarios. This increase in confidence was reported regardless of whether wearable data were reassuring or concerning; it points to the incomplete information practitioners currently experience after discharge, upon which practitioners are asked to make clinical decisions. Clinicians experience uncertainty regarding caregivers’ ability to assess their child’s recovery, and simple interventions to improve communication between the health care system and the caregiver reduce postoperative ED presentation by up to 50% [4,10]. Furthermore, with an enriched form of communication between the health care system, caregiver, and patient, it is anticipated that unnecessary ED presentation could be reduced even further.

It is important to note that while these results indicate the influence of wearable data on decision-making, it is not possible to determine, with certainty, from this study whether the addition of wearable data influenced the clinicians’ decision-making in a manner that can be delineated as correct. However, the changes seem to make clinical sense. Likewise, it is general practice for the institutions included in our study, and many others, that hemodynamically stable minor postoperative complications, such as a surgical site infection without systemic manifestations, be seen in the outpatient clinic if feasible to avoid the significant health care expenditure associated with the ED [6,12]. Moreover, how the likelihood of ED referral changed in response to the addition of concerning or reassuring wearable data did not differ between institutions. This supports consistency in wearable data interpretation across diverse practice settings and despite expected variation in institutional practice patterns.

Avoidable ED use has become an important focus of quality improvement initiatives to decrease unnecessary health care expenditures and health care saturation [6,13-15]. These initiatives were propagated by the adoption of digital health technology into clinical care. The momentum for this was largely propelled by the COVID-19 pandemic, during which the US Centers for Medicare and Medicaid (CMS) equated reimbursement of in-person and telemedicine visits, which was accompanied by the alignment of third-party payers [16]. As a result, many surgical departments implemented digital health platforms for postoperative patient care, which have been shown to be effective and efficient means of delivering care to children in the perioperative setting [17-24]. However, the objective data obtained during an in-person encounter remains largely absent; there are no vital signs available to interpret and the physical exam is limited to visual inspection [17]. Consumer-grade wearable devices, such as Fitbit, have been shown to supplant this absent objectivity by delivering measures of postoperative recovery including measures of heart rate, physical activity, and sleep [7,8].

Consumer wearable devices are unique in that they allow continuous capture and real-time transmission of health care measures which enables recovery trends to be examined [17]. When our survey participants were asked, 80% (20/25) to 96% (23/34) of clinicians reported they would reach out to the patient in response to concerning wearable data while only 24% (6/25) to 30% (7/23) would do so in response to reassuring wearable data. This demonstrates heart rate and step count data derived from wearable devices can be accurately analyzed and interpreted with ease by clinicians and can be integrated as a monitoring tool if wearable data are presented in real time. The integration of wearable data from Apple Health and Fitbit into the electronic health system has begun at several institutions [25]. Therefore, the practicality of wearables for postdischarge monitoring must be determined. This includes how data should be presented to optimize efficiency and how it will be incorporated into clinical workflow. Previous work has shown that clinicians favor data metrics familiar to them, such as heart rate, over those unique to wearable devices, such as step count [10]. Advances in wearable technology have continued to expand the range of measures available with the newest models including measures routinely used in practice, such as respiratory rate and oxygen saturation, which would further enhance clinician comfort and desirability of use.

### Limitations

This study has a number of limitations. First, the clinicians were responding to simulated patient scenarios. Although they were derived from actual patients, 1 set of wearable data was constructed for each scenario to create a pair of concerning and reassuring data. Second, clinicians respond to these questions in a simulation environment and survey format, which is low stakes and low stress in comparison with the high-demand workflow experienced by clinicians in daily practice. Prospective studies using actual patients are necessary to determine how wearable data change clinical decision-making in practice and their impact on postoperative outcomes and health care use. In addition, the Likert scales used for the survey were developed for the purposes of this study and have not been externally validated limiting the generalizability of our findings beyond this setting. Furthermore, the sites included in the study were all high-volume, academic children’s hospitals, and the study participants may not be representative of all clinicians caring for children after an appendectomy throughout the United States. Finally, the majority of respondents were attending surgeons. Although use in practice requires further elucidation, system patterns suggest it is more likely that nurse clinicians, advanced practice providers, and surgeons-in-training will field an initial postoperative telephone call. This further suggests the need to define the platform upon which wearable data will be implemented.

### Conclusion

Wearable data enhance the communication between caregivers, patients, and the health care team. The addition of objective measures of recovery to simulations of postoperative telephone scenarios impacts the recommendations made by pediatric surgery clinicians from diverse practice settings and improves clinician confidence when making remote patient assessments. Augmenting remote patient assessment offers the potential for improved triage of pediatric patients and could serve to reduce avoidable health care use. Furthermore, wearable devices, such as Fitbit, have the capability of providing real-time measures of recovery, which can be used as a postoperative monitoring tool to avoid delays in care for pediatric patients with postoperative complications.

## Abbreviations

CMS: Centers for Medicare and Medicaid
ED: emergency department
HIPAA: Health Insurance Portability and Accountability Act
